# Differential association of hypothyroidism with cardiovascular events and cardiovascular mortality: a systematic review and meta-analysis

**DOI:** 10.1007/s12020-026-04745-x

**Published:** 2026-08-15

**Authors:** Elisa Gatta, Andrea Delbarba, Enrico Vizzardi, Mario Rotondi, Carlo Cappelli

**Affiliations:** 1 Department of Clinical and Experimental Sciences, SSD Endocrinologia, University of Brescia, ASST Spedali Civili, Brescia, Italy; 2 Centro per la Diagnosi e Cura delle Neoplasie Endocrine e delle Malattie della Tiroide, University of Brescia, Brescia, Italy; 3 Institute of Cardiology, Department of Medical and Surgical Specialties, Radiological Sciences and Public Health, University of Brescia, Brescia, Italy; 4 Department of Internal Medicine and Therapeutics, University of Pavia, Pavia, Italy; 5 Istituti Clinici Scientifici Maugeri IRCCS, Unit of Internal Medicine and Endocrinology, Laboratory for Endocrine Disruptors, Pavia, Italy

**Keywords:** Hypothyroidism, Major cardiac events, Cardiovascular mortality, Myocardial infarction

## Abstract

**Purpose:**

The cardiovascular impact of hypothyroidism remains controversial, particularly regarding the distinction between cardiovascular disease occurrence and cardiovascular prognosis. We performed a systematic review and meta-analysis to separately evaluate the association between hypothyroidism and incident cardiovascular events and cardiovascular mortality.

**Methods:**

PubMed/MEDLINE, Scopus, and Web of Science were systematically searched for studies published between January 2014 and April 2026 evaluating cardiovascular outcomes in adult patients with overt or subclinical hypothyroidism compared with euthyroid controls. Separate random-effects meta-analyses were performed for cardiovascular events and cardiovascular mortality using pooled odds ratios (ORs) with 95% confidence intervals (CIs). Study quality was assessed using the QUIPS tool.

**Results:**

Nine studies involving 9,662 patients were included. Six studies evaluating cardiovascular events (5,795 participants) showed no significant association between hypothyroidism and incident cardiovascular events (OR 0.92, 95% CI 0.78–1.09; *p* = 0.35), with low-to-moderate heterogeneity (I² = 24.6%). By contrast, eight studies evaluating cardiovascular mortality (7,526 participants) demonstrated a significantly increased mortality risk among hypothyroid patients (OR 2.73, 95% CI 2.16–3.43; *p* < 0.0001), with low heterogeneity (I² = 9.7%). Leave-one-out sensitivity analyses confirmed the robustness and stability of the mortality findings. Qualitative synthesis consistently showed worse outcomes in acute and high-risk cardiovascular settings, including acute coronary syndromes, acute heart failure, spontaneous coronary artery dissection, and coronary artery bypass grafting.

**Conclusions:**

Hypothyroidism was more consistently associated with cardiovascular mortality than with incident cardiovascular events. However, the lack of a statistically significant association with cardiovascular events should not be interpreted as evidence of absence of effect. These findings are consistent with the hypothesis that thyroid dysfunction may be more closely associated with adverse cardiovascular outcomes after disease onset than with incident cardiovascular events.

**Supplementary Information:**

The online version contains supplementary material available at 10.1007/s12020-026-04745-x.

## Introduction

Hypothyroidism is one of the most common endocrine disorders worldwide and has long been associated with several mechanisms potentially involved in cardiovascular damage, including endothelial dysfunction, dyslipidaemia, oxidative stress, vascular stiffness, and chronic low-grade inflammation [1–5]. Thyroid hormones also play an important role in cardiovascular physiology by regulating myocardial contractility, vascular tone, and endothelial homeostasis [6]. Accordingly, thyroid dysfunction has traditionally been considered a potential contributor to cardiovascular disease development.

However, epidemiological evidence linking hypothyroidism to cardiovascular disease remains inconsistent. While several studies reported associations between thyroid dysfunction and surrogate markers of vascular damage, large observational cohorts and pooled analyses evaluating clinical cardiovascular outcomes yielded heterogeneous results, particularly for subclinical hypothyroidism [7–10]. In particular, previous studies reported inconsistent associations with myocardial infarction, coronary heart disease, stroke, and cardiovascular mortality after adjustment for age and traditional cardiovascular risk factors [8–11]. Moreover, the cardiovascular impact of hypothyroidism appears to vary according to age and clinical setting, with stronger associations often reported in younger and higher-risk populations [9, 11].

Recent evidence also suggests that hypothyroidism may exert different effects on cardiovascular disease occurrence and cardiovascular prognosis. In particular, thyroid dysfunction has been associated with impaired cardiovascular adaptation, reduced functional reserve, and worse outcomes in patients with established cardiovascular disease [6]. These observations raise the possibility that hypothyroidism may not behave as a classical atherogenic cardiovascular risk factor comparable to diabetes mellitus or hypertension, but rather as a modifier of cardiovascular vulnerability and prognosis.

Although several meta-analyses have examined the relationship between hypothyroidism and cardiovascular disease, most pooled incident cardiovascular events and mortality outcomes together or focused on a single endpoint. Consequently, whether hypothyroidism primarily influences cardiovascular disease occurrence or cardiovascular prognosis remains insufficiently clarified. Therefore, we performed a systematic review and meta-analysis to separately evaluate the association between hypothyroidism and incident cardiovascular events and cardiovascular mortality. The aim of the present study was to separately evaluate the association of hypothyroidism with cardiovascular event occurrence and cardiovascular mortality, and to explore whether these associations differ according to the cardiovascular outcome considered.

## Materials and methods

### Search strategy and inclusion criteria

A comprehensive literature search of the PubMed/MEDLINE, Scopus, and Web of Science databases was conducted to identify relevant studies evaluating the association between hypothyroidism and cardiovascular outcomes.

A review question was defined according to the Population, Exposure, Comparator, Outcome (PECO) framework: What is the association between hypothyroidism (exposure) and cardiovascular events or cardiovascular mortality (outcomes) compared with euthyroid individuals (comparator) in adult patients (population)?

The search strategy combined terms related to hypothyroidism and cardiovascular disease, including:

((“coronary artery disease“[MeSH Terms] OR “coronary heart disease” OR “acute coronary syndrome” OR “myocardial infarction” OR “coronary event*” OR “ischemic heart disease” OR “major adverse cardiovascular event*” OR MACE OR mortality OR “cardiovascular mortality”) AND (“hypothyroidism“[MeSH Terms] OR hypothyroidism OR “subclinical hypothyroidism” OR “thyroid dysfunction”)).

The search was restricted to studies published from January 1, 2014, to April 30, 2026, in order to focus on contemporary evidence and reduce potential variability related to historical differences in cardiovascular management, diagnostic approaches, and outcome reporting. Only articles published in English were considered. Reviews, editorials, conference abstracts, case reports, and preclinical studies were excluded. To minimize the risk of missing relevant studies, the reference lists of all included articles were also screened.

### Eligibility criteria

Studies were considered eligible if they evaluated adult patients with overt or subclinical hypothyroidism and reported cardiovascular outcomes compared with euthyroid controls. Eligible outcomes included coronary artery disease, acute coronary syndromes, myocardial infarction, other coronary or cardiac cardiovascular events, and cardiovascular mortality. Cerebrovascular outcomes, including ischemic stroke, were not specifically investigated. Control groups consisted of euthyroid individuals as defined by each study according to local thyroid function criteria. Reviews, editorials, letters, conference abstracts, and studies lacking an appropriate control group or sufficient outcome data were excluded.

### Study selection

C.C. and E.G. independently screened titles and abstracts identified through the search strategy. Full texts of potentially eligible studies were subsequently reviewed, and inclusion was determined according to predefined eligibility criteria. Disagreements were resolved through discussion and consensus.

### Reporting

**Fig. 1:**
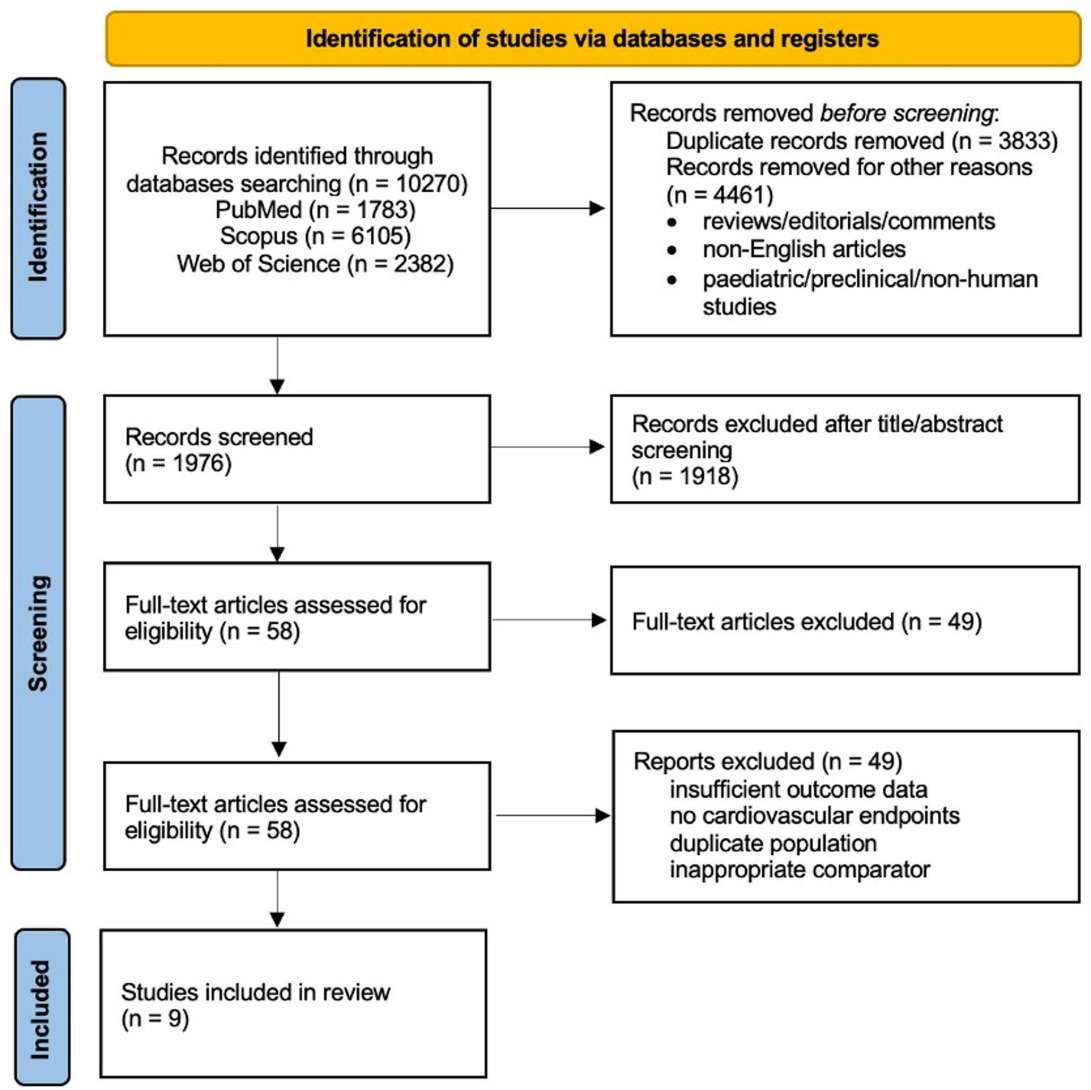
Flowchart of the study selection process for eligible studies

The protocol of this systematic review was registered in PROSPERO (CRD420261399128). Registration occurred after initiation of the review process and should therefore be considered retrospective. The study was conducted according to the Preferred Reporting Items for Systematic Reviews and Meta-Analyses (PRISMA) guidelines [12]. The corresponding PRISMA flow diagram is reported in Fig. 1. Study quality and risk of bias were assessed using the Quality In Prognosis Studies (QUIPS) tool [13].

### Data extraction

The reviewers independently extracted data from all included studies using full-text articles, tables, and supplementary materials. Extracted data included study characteristics (authors, publication year, country, and study design), patient characteristics (sample size, age, sex distribution, and cardiovascular setting), definition of hypothyroidism, number of cardiovascular events and deaths, and corresponding effect estimates. The main findings of the included studies are summarized in the Results section.

### Statistical analysis

Separate meta-analyses were performed for cardiovascular events and cardiovascular mortality. Dichotomous outcomes were pooled using odds ratios (ORs) with corresponding 95% confidence intervals (CIs). Random-effects models were specified a priori and applied to all analyses because clinical and methodological heterogeneity across studies was anticipated, including differences in cardiovascular settings, hypothyroidism definitions, outcome ascertainment, and study design. The choice of model was therefore based on expected between-study variability rather than on observed statistical heterogeneity. Statistical heterogeneity was assessed using the I² statistic and τ² estimates, with I² values > 50% considered indicative of substantial heterogeneity. Sensitivity analyses were performed using a leave-one-out approach to evaluate the robustness of the pooled estimates and the influence of individual studies. Publication bias was explored through visual inspection of funnel plots and, when appropriate (i.e., ≥ 10 studies), formally assessed using Egger’s regression test [14]. All analyses were conducted using RStudio (version 2025.05.1 + 513).

## Results

### Literature search

A total of 10,270 records were identified through the electronic literature search. After duplicate removal, 6,437 records remained. Following the application of predefined eligibility filters related to publication period, article type, language, and study population, 1,976 records underwent title and abstract screening. Subsequently, 58 full-text articles were assessed for eligibility, and nine studies were ultimately included [15–23] (Fig. 1). In detail, four studies were retrospective in nature, three were prospective cohort studies, one was a prospective multicentre observational study, and one was a prospective nationwide registry study. Most included studies originated from Europe (*n* = 4), followed by South America (*n* = 2) and Asia (*n* = 2), while one study was conducted in the United Kingdom.

Overall, risk of bias was generally low for outcome assessment and study attrition, whereas moderate concerns mainly emerged in domains related to confounding adjustment and variability in hypothyroidism definition. Differences in TSH thresholds, timing of thyroid function assessment, and adjustment strategies across studies represented the most relevant potential sources of bias. According to the QUIPS tool, the overall risk of bias was judged as low in four studies and moderate in five studies (Supplementary Table S1).

The main characteristics of the studies and their results are briefly presented in in Tables 1 and 2.

**Table 1:** Characteristics of the studies considered for the review

First author	Ref. *N*.	Year	Country	Study design	*N* Pts.	Sex M/F	Age
Soeiro	[15]	2018	Brazil	Observational retrospective study	505	297/208	62.9
Camacho Freire	[16]	2019	Spain	Observational cohort study	73	17/56	55 ± 12
De Matteis	[17]	2021	Italy	Retrospective study	1018	462/556	NA
Jabbar	[18]	2021	United Kingdom	Prospective multicentre observational study	1752	1287/465	63.8 ± 11.9
Wang	[19]	2021	China	Prospective cohort study	2569	1969/600	61.1 ± 11.7
Li	[20]	2022	China	Observational cohort study	909	615/294	NA
Camacho Freire	[21]	2025	Spain	Prospective nationwide registry study	389	45/344	54 ± 11
Jurin	[22]	2026	Croatia	Retrospective registry analysis	1584	1043/541	64 (56–72)
Martins	[23]	2026	Brazil	Retrospective cohort study	863	613/250	62.3 ± 9.0

Ref.: references; N.: number; Pts.: patients; NA: not available

**Table 2:** Results and main findings of the studies considered for the review

First author	Thyroid dysfunction definition	TSH values (Hypothyroid vs. Control)	Cardiovascular setting	Main outcome	Key findings
Camacho Freire [16]	Hypothyroidism history	NA	SCAD	Prevalence analysis	Higher prevalence of hypothyroidism among SCAD patients compared with ACS controls
Camacho Freire [21]	Hypothyroidism	NA	SCAD	Long-term outcomes	More aggressive angiographic phenotype and poorer long-term outcomes
De Matteis [17]	Overt hypothyroidism	9.20 (2.61–15.6) vs. 1.44 (0.89–2.26)	Acute heart failure	In-hospital mortality	Overt hypothyroidism independently associated with increased mortality
Jabbar [18]	Thyroid dysfunction spectrum	5.3 (4.5–6.5) vs. 1.8 (1.3–2.5)	Acute myocardial infarction	Composite cardiovascular outcomes	Thyroid dysfunction associated with adverse cardiovascular outcomes
Jurin [22]	Thyroid dysfunction	NA	Acute coronary syndrome	Short- and long-term mortality	Increased in-hospital, 30-day, and long-term mortality
Li [20]	Mild thyroid dysfunction	5.53 (4.66–6.89) vs. 1.32 (0.78–2.04)	STEMI	Cardiovascular prognosis	Mild thyroid dysfunction associated with worse prognosis
Martins [23]	Subclinical hypothyroidism	6.0 ± 1.73 vs. 1.82 ± 1.4	CABG	Major adverse cardiovascular events	Increased postoperative cardiovascular events after CABG
Soeiro [15]	Elevated TSH	NA	Acute coronary syndrome	In-hospital complications	Higher rates of cardiogenic shock and bleeding complications in patients with elevated TSH
Wang [19]	Hypothyroidism	4.88 ± 9.17 vs. 1.60 ± 1.04	Acute myocardial infarction	Heart failure and cardiovascular outcomes	Increased heart failure and adverse outcomes in hypothyroid patients

TSH: thyroid stimulating hormone; SCAD: spontaneous coronary artery dissection; ACS: acute coronary syndrome; STEMI: ST-segment elevation myocardial infarction; CABG: coronary artery bypass grafting; TSH: thyroid-stimulating hormone

### Qualitative analysis

Across the included studies, hypothyroidism was generally associated with worse cardiovascular outcomes compared with euthyroid subjects, although the magnitude of the association varied according to the clinical setting and the definition of thyroid dysfunction adopted by individual studies.

The most consistent associations emerged in acute cardiovascular settings. Across cohorts of patients with acute coronary syndromes (ACS) and acute myocardial infarction (AMI), hypothyroidism or elevated TSH levels were associated with higher rates of adverse in-hospital events, heart failure, and short- and long-term mortality [15, 19, 22]. In particular, Soeiro et al. reported increased rates of cardiogenic shock (13.6% vs. 6.1%, *p* = 0.029) and bleeding complications (15.3% vs. 6.5%, *p* = 0.012) among ACS patients with elevated TSH levels [15]. Similarly, Wang et al., in a large prospective AMI cohort, demonstrated higher rates of heart failure among hypothyroid patients (18.1% vs. 11.9%, *p* < 0.001), together with a significant association with composite cardiovascular outcomes [19]. More recently, Jurin et al. observed significantly increased in-hospital mortality (9.6% vs. 2.2%, *p* < 0.001), 30-day mortality (12.0% vs. 4.0%, *p* < 0.001), and long-term mortality (26.5% vs. 12.6%, *p* < 0.001) among ACS patients with thyroid dysfunction, with a more pronounced effect among women [22].

A prognostic role of hypothyroidism was also observed in other high-risk cardiovascular settings. De Matteis et al. found that overt hypothyroidism was independently associated with increased in-hospital mortality among patients hospitalized for acute heart failure [17]. Likewise, Martins et al. reported significantly higher rates of major adverse cardiovascular events following coronary artery bypass grafting (20.3% vs. 8.2%, *p* < 0.001) among patients with subclinical hypothyroidism compared with euthyroid individuals [23].

Studies investigating spontaneous coronary artery dissection (SCAD) further supported a possible relationship between hypothyroidism and vascular vulnerability. Camacho Freire et al. demonstrated a significantly higher prevalence of hypothyroidism among SCAD patients compared with matched ACS controls [16]. In a subsequent nationwide registry analysis, hypothyroid SCAD patients showed a more aggressive angiographic phenotype (19.0% vs. 9.0%, *p* = 0.044) and poorer long-term outcomes (27.0% vs. 11.0%, *p* = 0.033) compared with euthyroid individuals [21].

Overall, the qualitative analysis consistently suggested a stronger association between hypothyroidism and adverse prognosis in acute or high-risk cardiovascular conditions than with incident cardiovascular disease itself.

### Quantitative analysis

#### Cardiovascular events (angina/myocardial infarction)

**Fig. 2:**
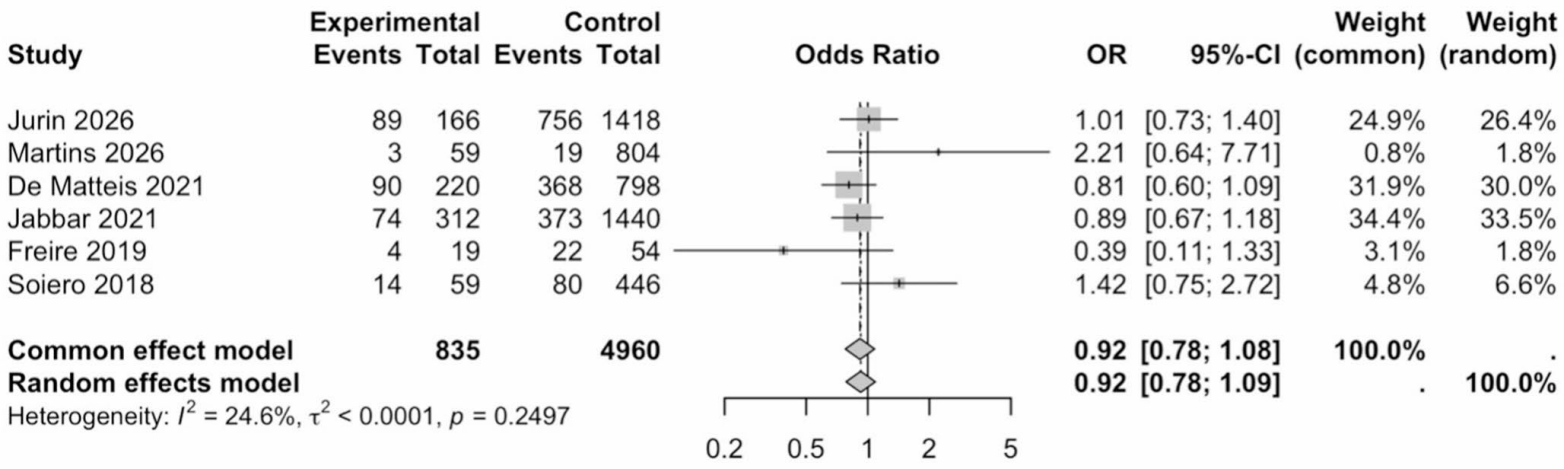
Forest plot of pooled effect estimates comparing major cardiac events in hypothyroid patients compared with controls

**Fig. 3:**
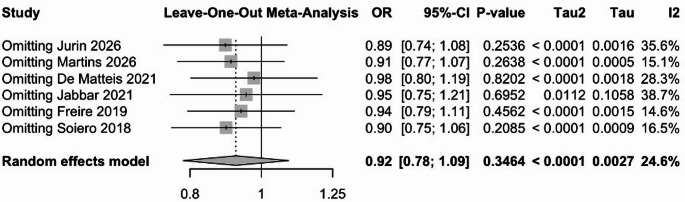
Funnel plot assessing publication bias in the meta-analysis comparing major cardiac events in hypothyroid patients compared with controls

**Fig. 4:**
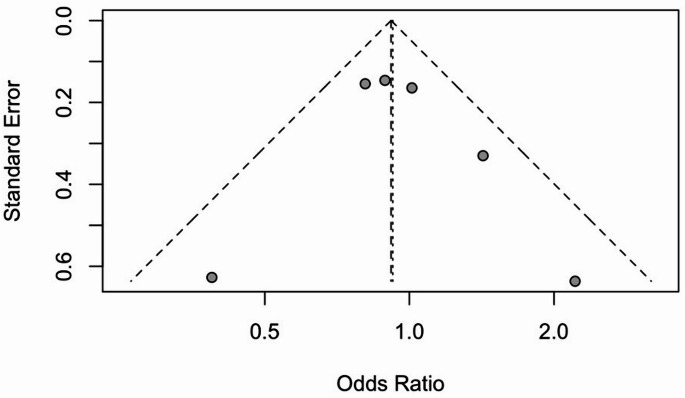
Leave-one-out sensitivity analysis of the pooled odds ratio of the meta-analysis comparing major cardiac events in hypothyroid patients compared with controls

A total of 6 studies were included [15–18, 22, 23], comprising 5,795 participants, of whom 835 were in the hypothyroid group and 4,960 served as controls. Overall, the random-effects meta-analysis did not demonstrate a significant association between hypothyroidism and coronary or cardiac cardiovascular events (OR = 0.92, 95% CI 0.78–1.09; *p* = 0.3464), with low-to-moderate heterogeneity (I² = 24.6%, τ² = 0.0027, *p* = 0.2497) (Fig. 2). Visual inspection of the funnel plot revealed effect estimates distributed around the line of no effect, with no consistent directional trend across studies (Fig. 3). Sensitivity analysis using a leave-one-out approach showed that the pooled effect size remained non-significant across all iterations (OR range: 0.89–0.98; all *p* > 0.05), with no individual study materially influencing the overall result. Heterogeneity varied modestly across iterations (I² range: 14.6%–38.7%), suggesting limited between-study variability and supporting the stability of the null finding (Fig. 4).

#### Cardiovascular mortality

**Fig. 5:**
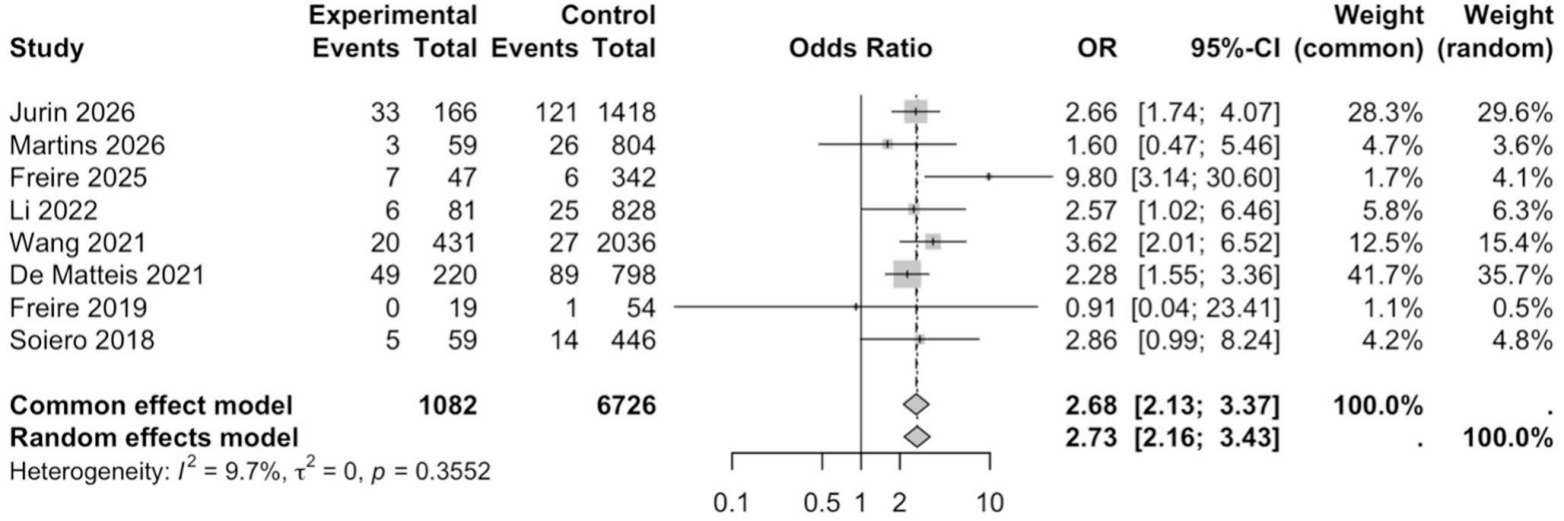
Forest plot showing pooled odds ratios for cardiovascular mortality in hypothyroid versus euthyroid patients

**Fig. 6:**
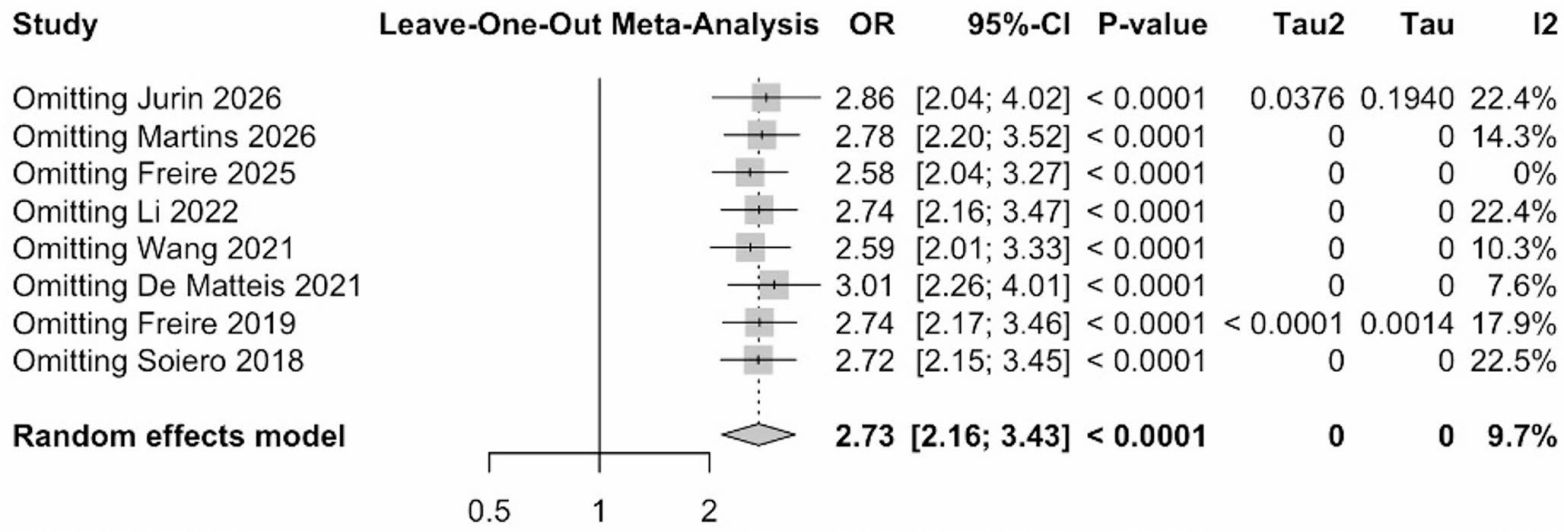
Funnel plot assessing publication bias in the meta-analysis comparing mortality for major cardiac events in hypothyroid patients compared with controls

**Fig. 7:**
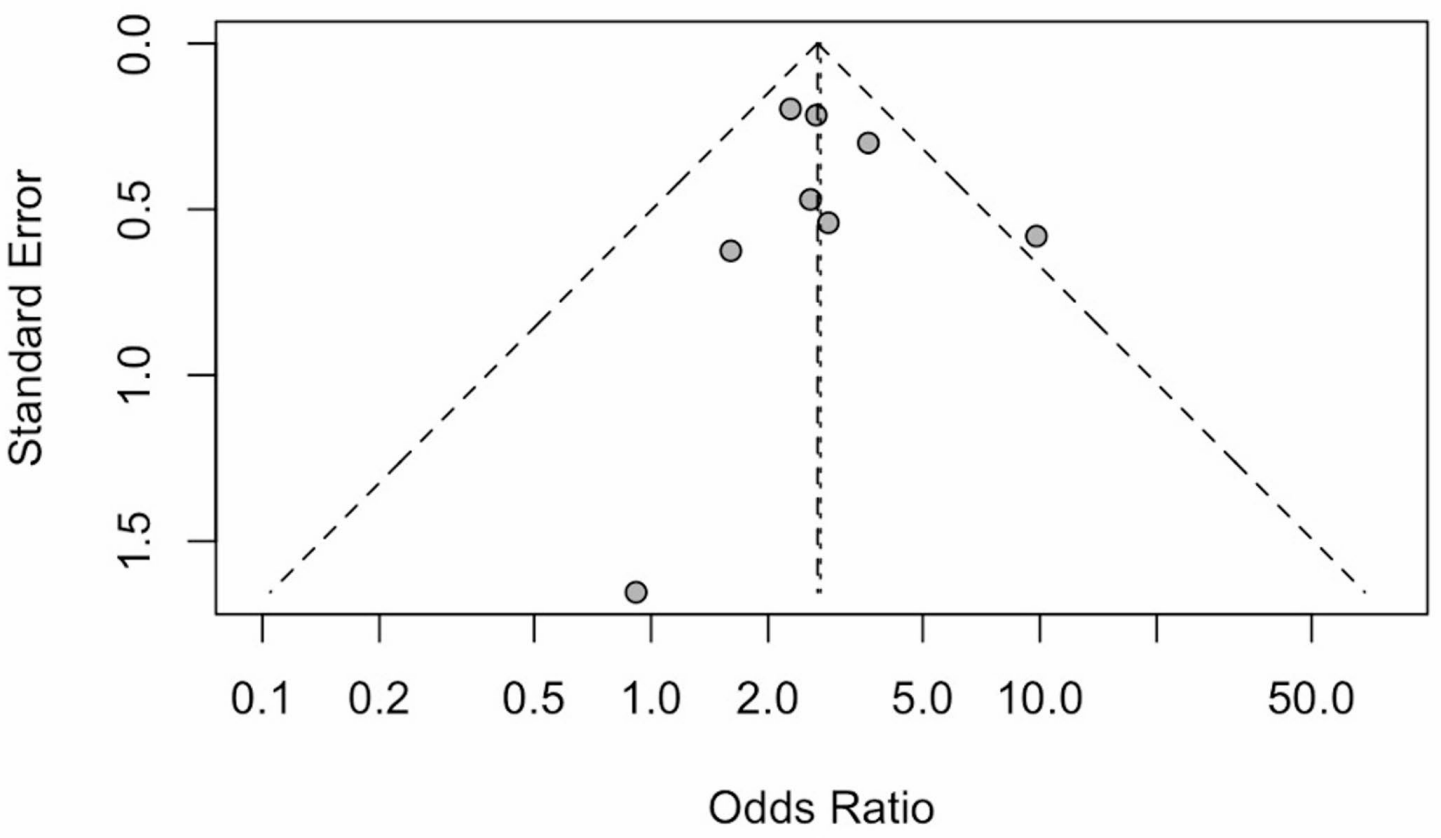
Leave-one-out sensitivity analysis of pooled cardiovascular mortality risk in hypothyroid versus euthyroid patients

A total of 8 studies were included [15–17, 19–23], comprising 7,526 participants, of whom 968 were in the hypothyroid group and 6,558 served as controls. Overall, the random-effects meta-analysis demonstrated a significantly higher risk of cardiovascular mortality in hypothyroid patients compared with controls (OR = 2.73, 95% CI 2.16–3.43; *p* < 0.0001), with low between-study heterogeneity (I² = 9.7%, τ² = 0, *p* = 0.3552) (Fig. 5). Visual inspection of the forest plot showed consistent effect sizes across studies, with all but one study favouring an increased risk in the hypothyroid group (Fig. 6). Sensitivity analysis using a leave-one-out approach confirmed the robustness of the findings, with pooled effect estimates remaining highly significant across all iterations (OR range: 2.58–3.01; all *p* < 0.0001). No single study exerted a disproportionate influence on the overall estimate. Heterogeneity remained low to moderate across iterations (I² range: 0%–22.5%), indicating a stable and consistent association across studies (Fig. 7).

Although both overt and subclinical hypothyroidism were included across studies, outcome data were inconsistently stratified according to thyroid phenotype. Consequently, separate quantitative analyses for overt and subclinical hypothyroidism could not be reliably performed.

## Discussion

The present meta-analysis was specifically designed to address a question that has received limited attention in previous quantitative syntheses: whether hypothyroidism exerts a similar impact on cardiovascular disease occurrence and cardiovascular prognosis. While several previous studies and meta-analyses evaluated the overall association between hypothyroidism and cardiovascular outcomes, most focused on individual cardiovascular endpoints or combined heterogeneous cardiovascular outcomes. Consequently, the relative impact of hypothyroidism on cardiovascular disease occurrence versus cardiovascular prognosis has remained incompletely understood. Our findings highlight a clinically relevant dissociation between these two domains, with hypothyroidism showing no significant association with coronary or cardiac cardiovascular event occurrence but a consistent association with cardiovascular mortality. This distinction may help reconcile some of the apparent inconsistencies reported in previous literature, where heterogeneous cardiovascular endpoints were frequently analysed together despite potentially reflecting different pathophysiological processes.

Hypothyroidism has long been associated with mechanisms potentially involved in cardiovascular damage, including endothelial dysfunction, impaired nitric oxide bioavailability, dyslipidaemia, oxidative stress, vascular stiffness, and chronic low-grade inflammation [24–28]. Previous mechanistic and vascular studies also suggested associations between thyroid dysfunction and surrogate markers of vascular remodelling, including carotid intima–media thickness and endothelial impairment [24, 26, 27]. However, epidemiological studies evaluating incident cardiovascular disease have yielded heterogeneous results. Large observational studies and pooled analyses reported inconsistent associations between subclinical hypothyroidism and myocardial infarction, coronary heart disease, or stroke, particularly after adjustment for age and traditional cardiovascular risk factors [5, 8, 9, 29]. The Thyroid Studies Collaboration showed that mild thyroid dysfunction was associated with modest or absent effects on incident cardiovascular disease in most populations, especially among older individuals [5]. Similarly, Razvi et al. reported a stronger association between subclinical hypothyroidism and ischemic heart disease in younger patients compared with elderly subjects [8]. Overall, these observations suggest that the association between hypothyroidism and incident cardiovascular disease may be weaker and less consistent than that observed for traditional cardiovascular risk factors such as diabetes mellitus or hypertension.

Although the association with incident cardiovascular events was not statistically significant, a different pattern emerged for cardiovascular mortality. This observation raises the possibility that the clinical consequences of hypothyroidism may become more apparent once cardiovascular disease is established rather than during the initial stages of disease development. While causality cannot be inferred from the available evidence, this hypothesis is supported by the more consistent findings observed in patients with acute and high-risk cardiovascular conditions.

By contrast, studies conducted in high-risk cardiovascular settings more consistently supported a prognostic role of thyroid dysfunction. Across the studies included in the present review, hypothyroidism was associated with worse outcomes in patients with acute coronary syndromes, acute myocardial infarction, acute heart failure, spontaneous coronary artery dissection, and coronary artery bypass grafting [3, 6, 21–23, 30–37]. These clinical observations provide a plausible context for interpreting the findings of the mortality meta-analysis, which demonstrated a consistent association between hypothyroidism and cardiovascular mortality across different cardiovascular settings.

The prognostic signal observed in our mortality analysis was remarkably robust. Hypothyroidism was associated with an almost threefold increase in cardiovascular mortality, with low heterogeneity and stable sensitivity analyses. Moreover, this association remained consistent across different cardiovascular settings, supporting the biological plausibility of a relationship between thyroid dysfunction and adverse cardiovascular prognosis. By contrast, the analysis of cardiovascular events did not demonstrate a statistically significant association between hypothyroidism and event occurrence. Although these findings may indicate a closer relationship between hypothyroidism and adverse cardiovascular outcomes after disease onset, the available evidence does not allow definitive conclusions regarding causality or the underlying mechanisms.

Several mechanisms may explain these findings. Thyroid hormones exert major effects on myocardial contractility, ventricular relaxation, endothelial homeostasis, vascular tone, and mitochondrial energetics [3, 18, 38]. Even mild thyroid dysfunction may impair cardiovascular reserve and reduce the ability to adequately respond to ischemic or hemodynamic stress. In this context, hypothyroidism may contribute less to plaque formation itself and more to impaired tolerance of cardiovascular injury once disease is established. Recent cardio-endocrine reviews further support this interpretation, suggesting that hypothyroidism may act predominantly as a cardiometabolic vulnerability amplifier rather than a direct initiator of atherosclerotic disease progression [4, 6].

This interpretation is also supported by studies evaluating thyroid hormone replacement therapy in cardiovascular populations. In patients with hypothyroidism and pre-existing cardiovascular disease, levothyroxine therapy was associated with reduced risks of major adverse cardiovascular events, cardiovascular hospitalization, and all-cause mortality [34]. Similarly, population-based data suggested a modest but significant reduction in cardiovascular events among treated patients with subclinical hypothyroidism [35]. Although these observational findings cannot establish causality, they support the hypothesis that restoration of thyroid hormone signalling may improve cardiovascular adaptation and resilience in vulnerable patients. From a clinical perspective, these findings support careful consideration of thyroid status in patients with established or acute cardiovascular disease, particularly when thyroid dysfunction is known or clinically suspected. However, the available evidence does not justify universal thyroid function screening after acute coronary syndrome or cardiovascular-specific levothyroxine targets; treatment should remain individualized, with cautious dose titration and avoidance of both under-replacement and iatrogenic thyrotoxicosis.

Another relevant aspect is the interaction between thyroid dysfunction, aging, and frailty-related pathways. Several studies included in the mortality analysis involved older and higher-risk cardiovascular populations, in whom hypothyroidism may represent not only a hormonal disorder but also a marker of impaired systemic adaptability. Reduced exercise tolerance, autonomic imbalance, endothelial dysfunction, sarcopenia, and chronic inflammation may all contribute to poorer recovery following acute cardiovascular events [36, 37]. This may partially explain why mortality outcomes appeared more consistently affected than incident cardiovascular events.

The present study has several strengths. First, unlike most previous reviews, cardiovascular events and cardiovascular mortality were analysed separately, allowing a more nuanced interpretation of the cardiovascular consequences of hypothyroidism and a clearer distinction between disease occurrence and disease prognosis. Second, the mortality analysis demonstrated low heterogeneity and excellent stability across sensitivity analyses. Third, the qualitative synthesis consistently supported a stronger association between thyroid dysfunction and adverse prognosis in high-risk cardiovascular settings than with incident cardiovascular disease itself.

Nevertheless, several limitations should be acknowledged. Most included studies were observational in nature, limiting causal inference. Definitions of hypothyroidism varied across studies, particularly regarding TSH thresholds and the distinction between overt and subclinical disease. A formal subgroup analysis according to overt versus subclinical hypothyroidism was not feasible because only a minority of studies reported outcome data separately by thyroid phenotype. The heterogeneity in hypothyroidism definitions across studies therefore represents an important limitation and may partly explain differences in effect estimates. In addition, the included studies enrolled patients across different cardiovascular settings, including acute coronary syndromes, acute myocardial infarction, acute heart failure, spontaneous coronary artery dissection, and coronary artery bypass grafting populations. Although statistical heterogeneity was low in both quantitative analyses, this clinical heterogeneity should be considered when interpreting the pooled estimates, as differences in disease severity, baseline cardiovascular risk, and underlying pathophysiological mechanisms may have influenced the observed associations. Information regarding levothyroxine treatment and adequacy of hormonal control was frequently unavailable. Future primary studies should adopt consistent biochemical definitions and TSH thresholds for overt and subclinical hypothyroidism, systematically report thyroid hormone replacement status and adequacy of biochemical control, and use standardized definitions of cardiovascular outcomes, particularly major adverse cardiovascular events. Such harmonized reporting would enable more reliable subgroup analyses according to thyroid phenotype, treatment status, and cardiovascular setting. Residual confounding related to age, frailty, cardiovascular therapies, and comorbidities cannot be excluded. Finally, the relatively limited number of studies prevented formal assessment of publication bias in some analyses.

In conclusion, hypothyroidism was more consistently associated with cardiovascular mortality than with incident coronary or cardiac cardiovascular events. These findings are consistent with the hypothesis that thyroid dysfunction may be associated with cardiovascular vulnerability, recovery capacity, and post-event adaptation. However, the observational nature of the available evidence precludes causal inferences, and residual confounding related to disease severity and patient characteristics cannot be excluded. From a clinical perspective, hypothyroidism may represent a potentially relevant marker of cardiovascular prognosis.

## Supplementary Information

Below is the link to the electronic supplementary material.

Supplementary Material 1
