# Supplementary material for: Differential association of hypothyroidism with cardiovascular events and cardiovascular mortality: a systematic review and meta-analysis
Source: Endocrine. 2026 Aug 15;91(1):277. doi: 10.1007/s12020-026-04745-x (PMC13477452; doi:10.1007/s12020-026-04745-x)
Supplement: Supplementary file 1 [file 12020_2026_4745_MOESM1_ESM.pdf]

Supplementary Table S1. Risk of bias assessment using the QUIPS tool

**Study**	**Study participation**	**Study attrition**	**Prognostic factor measurement**	**Outcome measurement**	**Confounding**	**Statistical analysis/reporting**	**Overall risk**
Soeiro et al., 2018	Moderate	Low	Moderate	Low	Moderate	Moderate	Moderate
Camacho Freire et al., 2019	Moderate	Low	Moderate	Low	Moderate	Low	Moderate
De Matteis et al., 2021	Moderate	Low	Moderate	Low	Moderate	Low	Moderate
Jabbar et al., 2021	Low	Low	Low	Low	Moderate	Low	Low
Wang et al., 2021	Low	Low	Low	Low	Moderate	Low	Low
Li et al., 2022	Moderate	Low	Moderate	Low	Moderate	Moderate	Moderate
Camacho Freire et al., 2025	Low	Low	Low	Low	Moderate	Low	Low
Jurin et al., 2026	Low	Low	Low	Low	Moderate	Low	Low
Martins et al., 2026	Moderate	Low	Moderate	Low	Moderate	Moderate	Moderate
